# Age-Related Tooth Wear Differs between Forest and Savanna Primates

**DOI:** 10.1371/journal.pone.0094938

**Published:** 2014-04-14

**Authors:** Jordi Galbany, Alejandro Romero, Mercedes Mayo-Alesón, Fiacre Itsoma, Beatriz Gamarra, Alejandro Pérez-Pérez, Eric Willaume, Peter M. Kappeler, Marie J. E. Charpentier

**Affiliations:** 1 Center for the Advanced Study of Hominid Paleobiology, Department of Anthropology, The George Washington University, Washington DC, United States of America; 2 Departament de Biologia Animal – Secció d'Antropologia, Universitat de Barcelona, Barcelona, Spain; 3 Departamento de Biotecnología, Universidad de Alicante, Alicante, Spain; 4 SODEPAL (Société du Parc d'Exploitation de la Lékédi)-COMILOG Society. Bakoumba, Gabon; 5 Behavioral Ecology & Sociobiology Unit, German Primate Center (DPZ), Göttingen, Germany; 6 Centre d'Ecologie Fonctionnelle et Evolutive UMR 5175, CNRS, Montpellier CEDEX 5, France; Monash University, Australia

## Abstract

Tooth wear in primates is caused by aging and ecological factors. However, comparative data that would allow us to delineate the contribution of each of these factors are lacking. Here, we contrast age-dependent molar tooth wear by scoring percent of dentine exposure (PDE) in two wild African primate populations from Gabonese forest and Kenyan savanna habitats. We found that forest-dwelling mandrills exhibited significantly higher PDE with age than savanna yellow baboons. Mandrills mainly feed on large tough food items, such as hard-shell fruits, and inhabit an ecosystem with a high presence of mineral quartz. By contrast, baboons consume large amounts of exogenous grit that adheres to underground storage organs but the proportion of quartz in the soils where baboons live is low. Our results support the hypothesis that not only age but also physical food properties and soil composition, particularly quartz richness, are factors that significantly impact tooth wear. We further propose that the accelerated dental wear in mandrills resulting in flatter molars with old age may represent an adaptation to process hard food items present in their environment.

## Introduction

Tooth morphology and enamel microstructure are linked to the mechanical properties of ingested food [1]–[6], and the effects of dental wear can be seen in occlusal surface morphology in particular [7]. Tooth wear is caused by a cumulative loss of enamel and dentine, principally due to the action of opposing teeth and the friction of hard and abrasive food objects [1] reflecting the interaction between feeding behavior and a species' environment. Tooth wear is also functionally significant because it is related to fitness components in several animal species. In koalas (*Phascolarctos cinereus*), for example, high rates of tooth wear are associated with an increase in the time spent feeding [8]. In roe deer (*Capreolus capreolus*), individuals showing more hypsodont teeth that wear at a lower pace present a longer life expectancy [9]. Finally, in sifakas (*Propithecus edwardsi*), age-related enamel tissue removal decreases individual nutrient intake efficiency, negatively affecting survival and reproduction [10]. There is, however, limited information on the impact of age on long-term enamel damage in animals that feed on food items with different physical properties that in turn vary according to micro-habitats and seasons [3], [11]–[14]. In experimental studies on vervet monkeys (*Chlorocebus aethiops*) using controlled diet, animals which feed on more abrasive food items present greater average annual tooth wear [15]. In wild howler monkeys (*Alouatta paliatta*), individuals exhibit faster rates of molar wear during the dry season [11]. Moreover, Ethiopian and Tanzanian baboons (*Papio hamadryas* and *P. cynocephalus*) show variation in wear rates according to differences in dietary ecology, ground cover and seasonal food availability [16]. Studies on 3D dental topography in wild howler monkeys (*Alouatta palliata*) [17], sifakas (*Propithecus edwardsi*) [10] and mountain gorillas (*Gorilla beringei beringei*) [18] show that tooth wear increases with age, and changes in molar crown occlusal morphology also affect the occlusal surface slope and relief as wear progresses. The shearing capacity in sifakas and mountain gorillas appears, however, to be independent of age [18], except in very old sifakas [10]. The fact that age seems not to greatly impact shearing capacity in these two species suggests that natural selection may shape tooth anatomy to maintain a certain degree of occlusal relief and functionality, especially in those folivorous primates that may need higher cusps for a lifelong mastication of tough fibrous foods [10], [18]. Other studies show that folivorous colobines present more sloping surfaces and more relief in tooth crowns than frugivorous cercopithecines at every tooth wear stage and age [4]. In line with this, some researchers have suggested that primate teeth have retained functionality after moderate wear, even improving it to a degree [19].

There are three main factors that affect tooth wear. First, hard items, such as fruits or parts of the pericarp around the seeds, require high bite forces to be fractured resulting in enamel cracks of the cervical and occlusal surfaces of molar teeth [1], [6], [20], [21]. Second, the amount of vegetal silica phytoliths has long been considered as an important agent responsible for tooth wear. Silica phytoliths may vary across plant parts [1], [22]. They are, for example, abundant in leaves and epidermis of flowering perennial evergreen plants [22]. Finally, food may also be covered with dust containing extraneous siliceous grit, including quartz or aluminosilicate minerals. These abrasive particles are present not only on the ground, but also in the canopy in both open habitats and tropical rain forests [1], [23], [24]. The relative importance of phytoliths and grit in causing tooth wear is still debated [25], [26]. Many authors suggest that silica phytoliths cause dental micro-scale indentation process in mammals [1], [21], [22], [27]. However, a study on the hardness of silica phytoliths found in four species of grass shows that they are considerably softer than tooth enamel, and therefore should not contribute to mammalian dental microwear as previously reported [28]. Alternatively, the authors propose that exogenous grit and dust are more likely causes of tooth wear [28]. In addition, recent research on the mechanisms behind tooth wear processes at a nanometer scale shows that quartz dust is a rigid abrasive, capable of fracturing and removing enamel pieces [26]. By contrast, phytoliths suffer deformation during their contact with the enamel, and form U-shaped grooves and flat troughs on enamel surface, but do not cause dramatic tissue loss [26]. In this study, Lucas and colleagues [26] conclude that dust containing mineral quartz appears to be the main wear agent of enamel during mastication. None of these current studies on animal tooth wear have, however, either analyzed or quantified the extrinsic particles found in the sediments where the studied individuals are living.

In order to determine whether the environment and feeding ecology are related to tooth wear variability in primates, we examine here the relationship between tooth wear and age in two African papionins, the forest-dwelling mandrills (*Mandrillus sphinx*) and the savanna-living yellow baboons (*Papio cynocephalus*) that show contrasting feeding behavior. Both species are semi-terrestrial and exhibit similar suites of cranio-dental features, including comparable enamel thickness [29]. Each species relies on different resources, however. Mandrills mostly feed on mechanically protected plant foods such as hard-shell fruits or seeds [30]. By contrast, baboons are highly exposed to exogenous grit and dust because they consume a large amount of underground storage organs (USOs) [24], [31], [32]. In particular, we compared tooth wear patterns, measured as the percent of dentine exposure (PDE), in these two species across ages and analyzed the composition of the soil in the two different habitats.

## Materials and Methods

### Ethics statement

This study complies with ethical protocols approved by the CENAREST institution (authorization number: AR0003/12/MENESRSIC/CENAREST/CG/CST/CSAR). The research adhered to the legal requirements of Gabon and Kenya, and to the American Society of Primatologists principles for the ethical treatment of nonhuman primates.

### Studied samples

The study population of mandrills (*Mandrillus sphinx*) inhabits the Lékédi Park, located 7 km northeast of the village of Bakoumba (Haut-Ogooué province) in Gabon [33] (see http://www.cefe.cnrs.fr/mandrillus/presentation). The landscape is mainly primary and secondary Marantaceae forest, with patches of humid open savannas. The average annual rainfall is 1,474 mm with a long dry season that spans from June to September [34].

**Figure 1:**
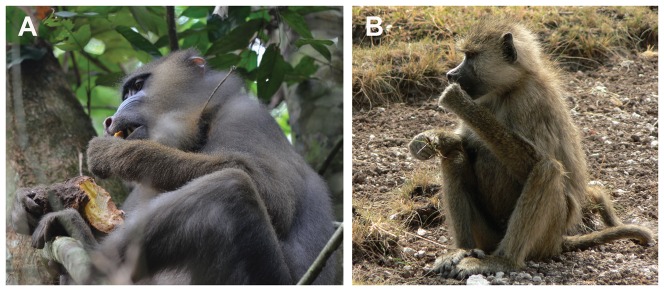
A mandrill from the Lékédi Park (A) and a baboon from Amboseli (B) feeding on roots and corms, respectively. Both individuals are males.

Mandrill is a semi-terrestrial forest-dwelling species that spends much of its daily activity foraging through leaf litter on the forest floor [30], [35]. The studied population of mandrills initially included 65 captive individuals of both sexes and all ages housed at the CIRMF (Centre de Recherches Médicales de Franceville, Gabon) that were released on two different occasions (2002 and 2006) [33]. At the time of this study (April 2013), the habituated mandrill population numbered around 100 individuals, and about 80% of them were wild-born individuals. We were able to easily track this habituated group of mandrills by following four adult females fitted with radio-collars [33]. All individuals from this population forage near-continuously throughout the day, feeding mainly on hard fruits and seeds from the ground (75% of food eaten year-round) [34] (Figure 1A).

We captured and analyzed a subset of 37 mandrills of both sexes, aged from 3 to 19.6 years. Twenty-three of them were wild-born animals and their estimated ages were assigned using body, skin and fur condition, dental eruption pattern in juveniles [36] and previous experience with known-aged mandrills. The other 14 animals were captive-born individuals housed at the CIRMF that were later released into the park at different ages (Table S1).

The yellow baboon (*Papio cynocephalus*) study population inhabits a semi-arid, short-grass savanna ecosystem in the Amboseli basin (southern Kenya) at the northwestern base of Mt. Kilimanjaro. This population has been intensively studied for almost four decades [37] (see http://amboselibaboons.nd.edu). Amboseli is one of the driest habitats of baboons. The average annual rainfall is very low (348 mm), with a seasonal pattern ranging from 150 to 500 mm. A long dry season occurs from June to October during which fruits and forbs are scarce. However, the baboons' diet shows relative stability across seasons. Underground Storage Organs (USOs), such as grass and sedge corms, fruits and blade bases, are the principal food resources for baboons and are consumed year round [38] (Figure 1B). This study included 95 individuals captured between 2006 and 2008 [31]. Dates of birth are known for most individuals within a range of a few days, and for those individuals without associated records, we estimated ages based on physical growth and development [39].

Our sample includes mandibular and maxillary M1-M2 tooth casts of mandrills (*n* = 37 M1; *n* = 32 M2) and baboons (*n* = 94 M1; *n* = 94 M2). In the mandrill population, animals were darted using blowpipes and briefly immobilized (∼30 sec) with a mix of two anaesthetics (400 mg ketamine for 500 mg of xylazine). Animals were then woken using atipamezole (Antisedan ND, 0.5 mg/ml). In Amboseli baboons, we used similar methods but animals were anesthetized with Telazol [31].

Morphological and physiological data were collected from individuals prior to obtaining high quality tooth molds. Postcanine molar crowns (maxilla and mandible) were washed and brushed slightly and molded using Coltène *Speedex* dental impression material. Resultant replicas were produced at University of Barcelona using polyurethane [31], [40] and prepared for further morphological analysis.

### Tooth wear analysis

**Figure 2:**
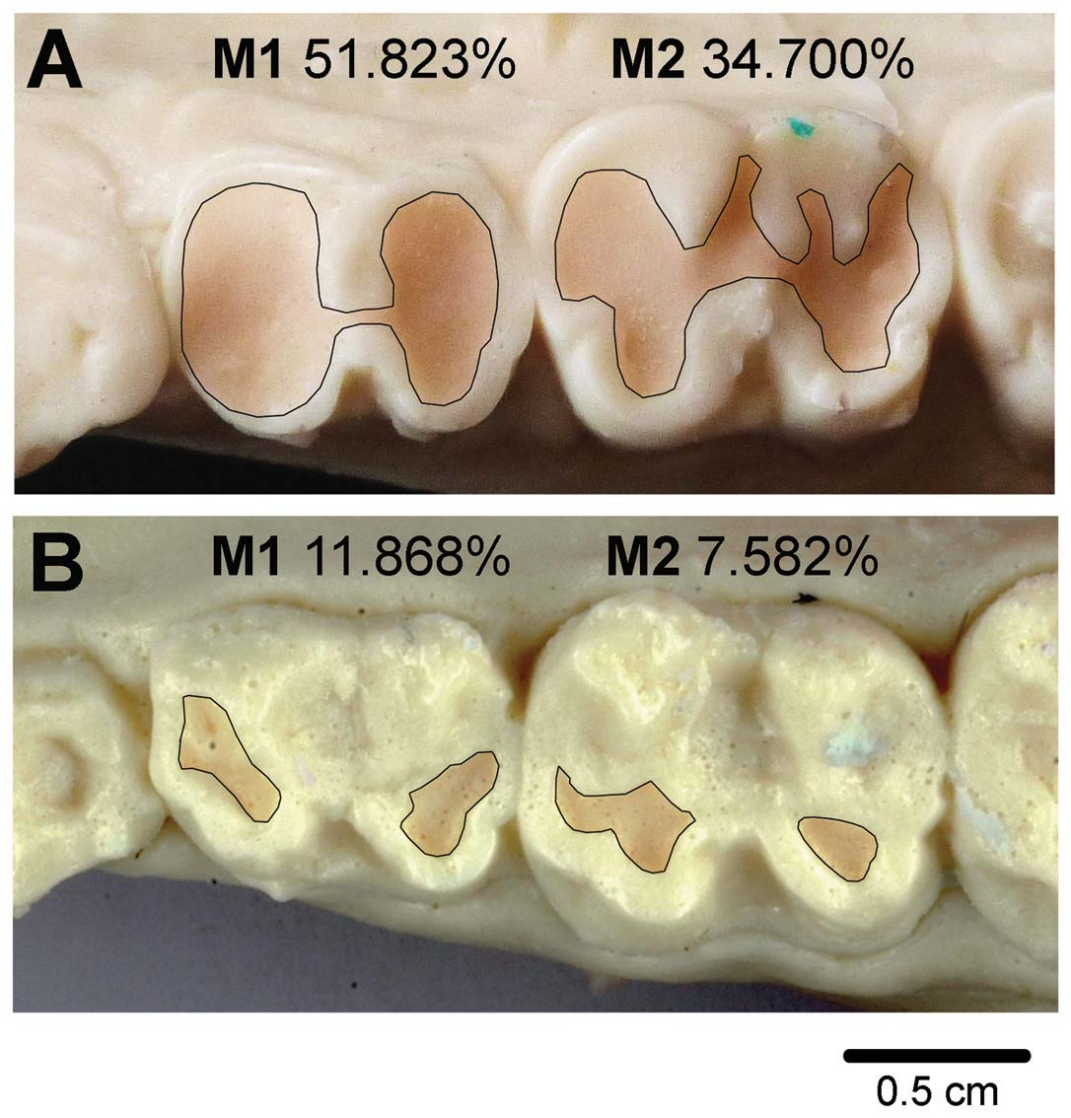
Mandibular molar occlusal images showing tooth crown of similar ages with different percent of dentine exposure (PDE). (A) Male mandrill “33” aged 11, and (B) male baboon “Amok” aged 12. Outline of the dentine areas are shown (see Material and Methods section for details on the analysis on tooth wear).

Occlusal digital photographs were taken and analyzed using ImageJ [41] to obtain the percent of dentine exposure (PDE) for each upper and lower M1-M2 molars (Figure 2) following previously established standard procedures [31]. An average PDE (upper and lower) was obtained for each tooth and studied animal. When missing or broken tooth were found, PDE was based on the available molar (see Table S1; see ref. [31] also for raw data in baboons).

Results from linear regressions between PDE and age were complemented with an overall multivariate analysis of variance (MANOVA) to detect changes with age for each molar tooth in both species and analysis of covariance (ANCOVA) to test for the homogeneity of slopes between species (mandrills vs. baboons), sex (female vs. male) and origin (wild vs. captive born) in the case of mandrills. Analyses were conducted using PAST [42] and SMATR (Standardized Major Axis Tests and Routines) software [43].

### Soil composition analysis

Regarding the sediment analyses, we collected soil samples in both Amboseli (*n* = 9) and the Lékédi Park (*n* = 7) locations, in the area where the primate populations live year round. The samples were pulverized and the mineral composition was determined using X Ray Diffraction analysis (XRD) (Bragg-Brentano PANalytical X'Pert PRO MPD alpha1), following the standard Powder Diffraction File of the ICDD-JCPDS (International Centre for Diffraction Data – Joint Committee of Powder Diffraction Standards). All the analyses were done at the XRD Unit of the Centres Científics i Tecnològics at the University of Barcelona. Statistical differences in mineral composition (t-test) were performed in PAST [42].

## Results

Cross-sectional linear and quadratic regressions of PDE with age in mandrills and baboons for M1 and M2 teeth yielded highly significant positive relationships (*p*<0.001 in all cases), although quadratic regressions were always more informative, following the Akaike information criterion (AIC) (Table 1). When logarithmic and arcsin transformations (for age and PDE, respectively) were used, the resulting linear regressions were also significant (*p*<0.001 in all cases) in mandrills (M1: R^2^ = 0.861; M2: R^2^ = 0.871) and baboons (M1: R^2^ = 0.672; M2: R^2^ = 0.770). An overall MANOVA test (Table 2) revealed that PDE changed with age within molar teeth for mandrills (R^2^ = 0.784, Wilkś λ = 0.170, F_2,29_ = 70.82, *p*<0.0001) and baboons (R^2^ = 0.736, Wilkś λ = 0.224, F_2,90_ = 156.4, *p*<0.0001).

**Table 1:** Quadratic (Q) and linear (L) regressions for predicted percent of dentine exposure (PDE) with age for M1 and M2.

		Tooth	n	R^2^	F	*p*	AIC	Equation
	Q	M1	37	0.849	95.703	**<0.001**	1342.4	PDE = -0.069*age^2^+4.521*age-14.675
Mandrills		M2	32	0.742	41.803	**<0.001**	896.4	PDE = 0.032*age^2^+1.376*age-6.902
	L	M1	37	0.839	182.004	**<0.001**	1426.2	PDE = 3.125*age-9.189
		M2	32	0.738	84.482	**<0.001**	911.97	PDE = 2.061*age-9.941
	Q	M1	94	0.856	269.770	**<0.001**	1311.1	PDE = 0.123*age^2^-1.554*age+8.557
Baboons		M2	94	0.769	151.093	**<0.001**	1306.9	PDE = 0.072*age^2^-0.591*age+1.940
	L	M1	94	0.756	284.614	**<0.001**	2212.7	PDE = 1.904*age-12.182
		M2	94	0.713	228.282	**<0.001**	1618.4	PDE = 1.446*age-10.299

Significant differences are shown in bold (*p*<0.05). AIC: Akaike Information Criterion.

**Table 2:** Multivariate regressions of percent of dentine exposure (PDE) with age (M1 and M2) in mandrills and baboons.

Mandrills	Variable	Slope	Error	Intercept	Error	R^2^	*p*
	PDE M1	3.1553	0.28368	−9.552	3.0259	0.805	<0.0001
	PDE M2	2.0606	0.22419	−9.941	2.3913	0.738	<0.0001

MANOVA	R^2^	MSE	Wilks′ λ	df1	df2	F	*p*
	0.784	39.57	0.170	2	29	70.82	<0.0001

Baboons	Variable	Slope	Error	Intercept	Error	R^2^	*p*
	PDE M1	1.9027	0.11392	−12.161	1.3573	0.754	<0.0001
	PDE M2	1.4348	0.09708	−10.206	1.1567	0.706	<0.0001

MANOVA	R^2^	MSE	Wilks′ λ	df1	df2	F	*p*
	0.736	20.94	0.224	2	90	156.4	<0.0001

Significant differences are shown in bold (*p*<0.05).

We performed slope comparisons to determine the differences in tooth wear with age in relation to sex (for similar results on baboons see ref. [44]). In this analysis, mandrills did not present significant between-sex differences in the slope for M1 and M2. As the mandrill population was constituted by wild-born and captive-born individuals (Table S1), tooth wear according to an animal's origin was also analyzed. We found no significant differences in tooth wear between wild and captive origin of the studied mandrills for both M1 and M2, although for M2 the relationship was close to significance (*p* = 0.053) (Table 3).

**Table 3:** Slope comparisons of tooth wear (M1 and M2) according to the species, the sex and the origin of the studied individuals (for mandrills).

Comparison	Tooth	n	Common Slope	SMATR Test	*p*
Mandrills vs. baboons	M1	131	2.498	23.229	0.001
	M2	126	1.857	8.711	0.005
Females vs. males (mandrills)	M1	37	3.456	0.218	0.634
	M2	32	2.415	1.606	0.174
Wild vs. captive born (mandrills)	M1	37	3.691	1.024	0.304
	M2	32	2.540	3.781	0.053

Significant differences are shown in bold (*p*<0.05).

**Figure 3:**
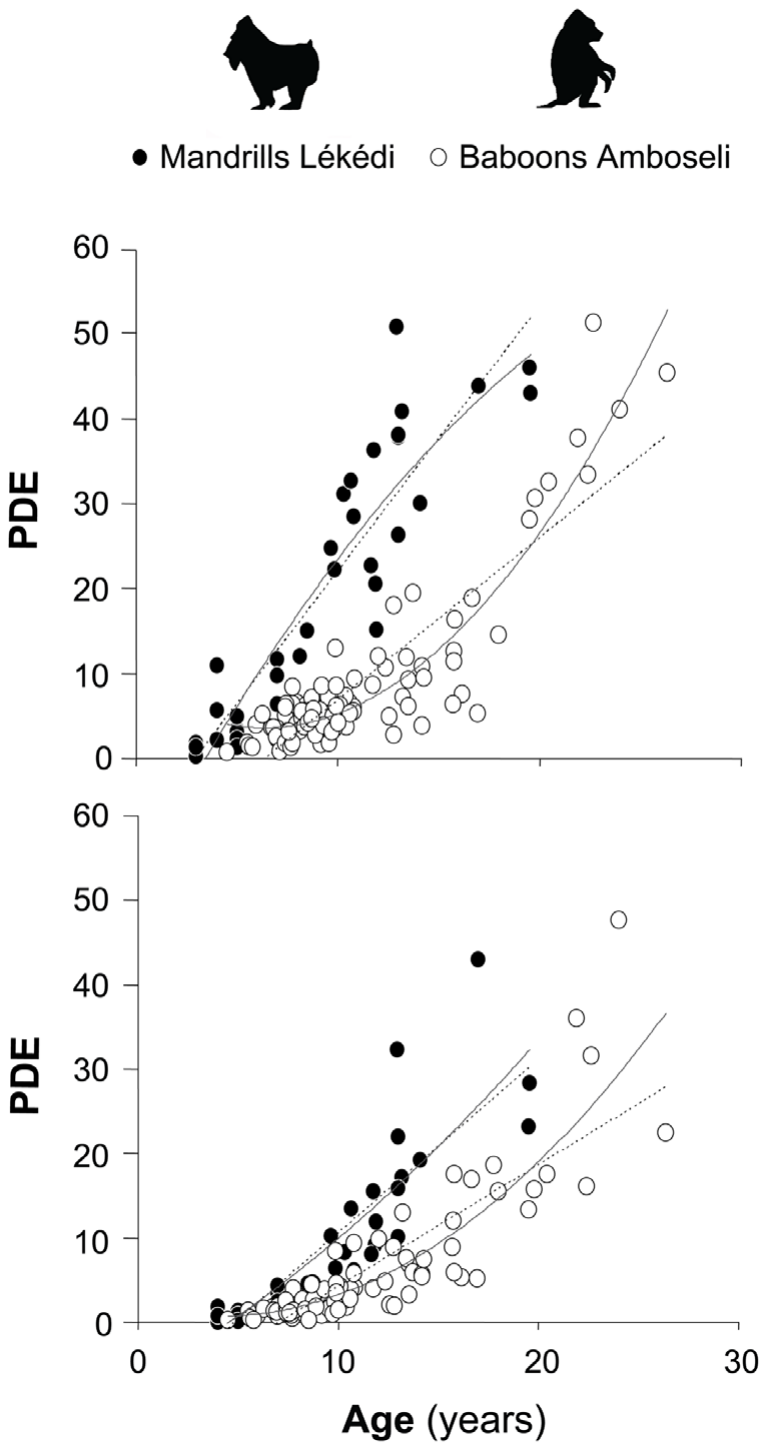
Quadratic (continuous lines) and linear (dashed lines) regressions for predicting percent of dentine exposure (PDE) with age in mandrills and baboons M1 (A) and M2 (B).

We found significant interspecific differences in the homogeneity of slopes for both M1 (*p* = 0.001) and M2 (*p* = 0.005), indicating a more rapid increase in PDE with age in mandrills compared to baboons (Figure 3 and Table 3). For example, for the linear regression of M1, the PDE of a 10-year-old baboon is 31% of the value for a mandrill of the same age (6.9% vs. 22.1%). This comparison is 43% (16.4% vs. 37.7%) when comparing 15-year-old individuals.

Finally, sediment analyses showed different mineral compositions according to the location of origin. Sediments from Amboseli showed a mineral composition mostly composed of calcite, dolomite and ankerite, sepiolite clays, feldspartz, quartz and also an amorphous phase. In contrast, sediments found in the Lékédi park were composed mostly by kaolinite, an amorphous phase and gibbsite, and also quartz and undetermined clay. Quantitative analyses of quartz, the only mineral found in the analyzed samples capable to abrade tooth enamel [26], revealed that Amboseli sediments presented (mean±SD) 1.49%±0.89 of quartz and the Lékédi park sediments showed 7.87%±1.69 of quartz (F = 2.26, *p*<0.0001).

## Discussion

As in other wild primates [10], [18], we found that tooth wear increases with age in mandrills and baboons. In both species, the difference in tooth wear between M1 and M2 likely reflects earlier tooth eruption and functionality for M1 [36], [45].

Sexes did not differ in tooth wear for both M1 and M2 in the two species (see also [44] for baboons). In mandrills, the animal's origin did not influence tooth wear in both M1 and M2, although for M2, the relationship was close to significance: wild-born mandrills tend to present higher tooth wear rates, measured as PDE. However, most of the captive born mandrills lived only a few years in captivity. Only two mandrills were released in the wild at 9.5 and 9.6 years old, and were captured for this study when they were 19.5 and 19.6 years old, respectively. The remaining individuals were released in the Lékédi Park when they were still young animals with deciduous teeth (Table S1). Moreover, all captive-born individuals lived in naturalistic enclosures at the Centre international de Recherches Médicales de Franceville (Gabon, CIRMF) and were probably exposed to similar food items as the ones they are experiencing in wild conditions [33]. Finally, removing these two individuals from our analyses did not change our results (data not shown).

Sex and origin homogeneity allowed us to compare both species, and we found a clear interspecific variation. Mandrills showed higher age-related tooth wear than baboons as well as an accelerated rate of wear with age. These results suggest that there are differences in physical properties of foods and abrasive particles consumed by the two species. Field data indicate that both species are eclectic omnivores, but they rely on different resources. Forest-dwelling mandrills feed year-round a high percentage (>70%) on hard fruits and seeds from the ground [34]. By contrast, Amboseli baboons rely heavily on underground storage organs (USOs) by mostly digging up grass corms (30% of food eaten year-round). During the long dry season, when the availability of fruits, grass blades and forbs is constrained, USOs represent up to 60% of baboon's diet [38]. Thus, both species show rather different dietary strategies, affecting molar enamel wear differently [2].

The protective casing of nuts or fruit exocarps consumed by mandrills requires very high bite force magnitudes to be processed, increasing the risk of enamel tooth fractures [1], [5], [6], [10], [20], [46], [47]. Moreover, the quartz load found in the sediments of Lékédi Park is on average more than 5 times greater than that of Amboseli. Because food resources are covered by soil and dust from the sediments, mandrills are exposed to a higher amount of mineral quartz and probably ingest higher proportion of this abrasive grit.

Mandrills should be well adapted to process hard food items because they exhibit narrow dental arcades and large symphyses involving a high adductor force [48]. They also possess expanded premolars [49]. However, large-scale hardcover objects (indenter radius; *r*_i_ *= *2 to 20 mm), that mandrills consume year-round [35], require forces stronger than 1kN to breakdown [1], [6], [20]. This extreme chewing force together with the high proportion of extrinsic quartz present in the environment of mandrills likely result in fast enamel cracking and early dentine exposure [20], [25]. A similar case was found in wild *Lemur catta* from Beza Mahafaly, where animals need an extreme chewing force to break the *Tamarindus* fruits, resulting in fast enamel cracking [13], [50]. By contrast, baboons exhibit lower tooth wear rates than mandrills, which may be primarily caused by extrinsic quartz from the soil, and possibly by the presence of plant silica phytoliths [31]. A lower proportion of grit, which comprises very small-scale hard object indenters (*r*_i_ *= *5 to 50 µm), implies that the teeth wear more slowly because these abrasives cause cumulative damage to enamel but not enamel cracking [2] as in the case for mandrills.

The link between molar occlusal topography and food mechanical properties is crucial for food breakdown [1], [7], [51]. Both mandrills and baboons show dental morphological similarities in sharp cusp pattern, hence tooth wear differences may not be explained by dental occlusal topography in these species. Theoretically, thick enamel should benefit species like mandrills by extending tooth life through a protection against large-scale fractures [52]. Intriguingly, mandrills present enamel thickness similar to baboons [29]. Although mandrills wear teeth more quickly than baboons, they could retain their functionality [19]. Indeed, flatter worn teeth present a lower crown relief that could be more efficient for hard food items because a uniform distribution of high occlusal forces is better to process hard objects [29], [53]. Teeth with sharper cusps can create higher stress concentrations than dull cusps, which are more efficient for fracturing brittle food items [46]. Models on the interaction between molar occlusal morphology and food properties [54] predict that sharp cusps produce much higher tensile stress ratios in enamel than in brittle food items. Thus, the differences in food mechanical properties could explain the variation in wear rates found between baboons and mandrills (but see ref. [46] for a discussion about the lack of efficiency of low-crowned molars to process hard objects in hominin models). If so, the present study contributes to the hypothesis that primate teeth wear in a manner that keeps them mechanically efficient for processing specific foods. Although a high percent of dentine exposure may not be adaptive *per se*, especially in younger animals, it should represent a response to a given environment and feeding ecology that maintain functionality. In order to determine to what extent the differences in tooth wear rates are adaptive, data on fitness are now needed as well as long-term *in vivo* research on well-known primate populations.

## Supporting Information

Table S1 **Captured mandrills, sex, wild (W) or captive born (C), date of release of captive born individuals, darting date and darting age, and percent of dentine exposure (PDE) for each molars (M_1_: lower M1; M^1^: upper M1; M_2_: lower M2; M^2^: upper M2).** (I) Molar was not yet erupted or only partially erupted; (II) Molar cast was of insufficient quality. NA: not applicable.
(DOC)
