# Supplementary material for: Age-Related Tooth Wear Differs between Forest and Savanna Primates
Source: PLoS One. 2014 Apr 14;9(4):e94938. doi: 10.1371/journal.pone.0094938 (PMC3986402; doi:10.1371/journal.pone.0094938)
Supplement: Table S1 — Captured mandrills, sex, wild (W) or captive born (C), date of release of captive born individuals, darting date and darting age, and percent of dentine exposure (PDE) for each molars (M1: lower M1; M1: upper M1; M2: lower M2; M2: upper M2). (I) Molar was not yet erupted or only partially erupted; (II) Molar cast was of insufficient quality. NA: not applicable. (DOC) [file pone.0094938.s001.doc]

**Supplementary Information**

**Table S1.** Captured mandrills, sex, wild (W) or captive born (C), date of release of captive born individuals, darting date and darting age, and PDE for each molars (M_1_: lower M1; M^1^: upper M1; M_2_: lower M2; M^2^: upper M2). (I) Molar was not yet erupted or only partially erupted, (II) Molar cast was of insufficient quality. NA: Not applicable.

| **Animal ID** | **Sex** | **W/C** | **Date of release** | **Age at release** | **Darting date** | **Darting Age** | **M_1_** | **M^1^** | **M_2_** | **M^2^** |
| --- | --- | --- | --- | --- | --- | --- | --- | --- | --- | --- |
| 3 | f | W | NA | NA | 22/09/2012 | 3 | 1.100 | 1.410 | (I) | (I) |
| 6 | f | W | NA | NA | 22/09/2012 | 3 | 1.735 | 1.781 | (I) | (I) |
| 7 | f | C | 01/10/2002 | 9.55 | 17/09/2012 | 19.52 | 59.402 | 32.562 | 30.550 | 15.854 |
| 13 | f | C | 01/09/2006 | 5.70 | 25/09/2012 | 11.77 | 36.232 | (II) | 15.498 | (II) |
| 16 | f | C | 01/09/2006 | 2.46 | 17/09/2012 | 8.51 | 23.884 | 6.020 | 4.561 | (II) |
| 18 | f | W | NA | NA | 22/09/2012 | 4 | 5.597 | (II) | 0.721 | (I)/(II) |
| 19 | f | C | 01/10/2002 | 4.56 | 11/04/2012 | 14.09 | 35.562 | 24.544 | 28.630 | 9.736 |
| 20 | f | W | NA | NA | 11/04/2012 | 9.87 | 25.492 | 18.952 | 9.389 | 3.212 |
| 21 | f | W | NA | NA | 14/04/2012 | 12.94 | 51.243 | 50.304 | 42.171 | 22.492 |
| 23 | f | C | 01/09/2006 | 3.60 | 19/09/2012 | 9.65 | 37.366 | 12.185 | 13.511 | 6.855 |
| 25 | f | C | 01/09/2006 | 5.80 | 25/09/2012 | 11.87 | 30.719 | 10.347 | 12.380 | 5.979 |
| 29 | f | C | 01/10/2002 | 3.65 | 15/04/2012 | 13.19 | 38.819 | 42.883 | 19.586 | 14.755 |
| 30 | f | C | 01/09/2006 | 4.25 | 24/09/2012 | 10.32 | 43.937 | 18.301 | 11.774 | 4.882 |
| 31 | f | W | NA | NA | 19/09/2012 | 7 | 7.884 | 4.764 | 4.247 | 2.775 |
| 34 | f | W | NA | NA | 15/04/2012 | 3 | 0.182 | (I) | (I) | (I) |
| 38 | f | C | 01/10/2002 | 0.67 | 21/09/2012 | 10.65 | 30.564 | 34.699 | 15.089 | 11.799 |
| 39 | f | W | NA | NA | 19/09/2012 | 5 | 5.041 | 1.204 | 1.347 | (I) |
| 40 | f | W | NA | NA | 19/09/2012 | 7 | 11.256 | 8.152 | 3.574 | 1.169 |
| 41 | f | W | NA | NA | 22/09/2012 | 5 | 4.901 | (II) | (I) | (I)/(II) |
| 42 | f | W | NA | NA | 21/09/2012 | 3 | 2.063 | 0.602 | (I) | (I) |
| 44 | f | C | 01/10/2002 | 9.59 | 17/09/2012 | 19.56 | 45.496 | 40.333 | 35.072 | 21.613 |
| 46 | f | W | NA | NA | 21/09/2012 | 5 | 2.635 | 2.034 | 0.824 | (II) |
| 48 | f | C | 01/09/2006 | 5.85 | 20/09/2012 | 11.91 | (II) | 15.146 | 17.977 | 5.736 |
| 56 | f | C | 01/10/2002 | 1.67 | 22/09/2012 | 11.65 | 20.444 | 24.928 | 9.937 | 6.170 |
| 4 | m | W | NA | NA | 21/09/2012 | 5 | 3.397 | 0.700 | 2.089 | 0.402 |
| 8 | m | W | NA | NA | 17/09/2012 | 4 | 2.479 | 1.623 | 0.590 | 0.268 |
| 9 | m | W | NA | NA | 24/09/2012 | 4 | 5.561 | 16.383 | (II) | 1.801 |
| 12 | m | W | NA | NA | 18/09/2012 | 5 | 3.024 | 3.640 | 1.489 | 1.481 |
| 17 | m | W | NA | NA | 15/04/2012 | 4 | 2.887 | 1.434 | 0.000 | (I) |
| 33 | m | W | NA | NA | 13/04/2012 | 13 | 44.020 | 31.489 | 34.700 | 9.308 |
| 35 | m | W | NA | NA | 13/04/2012 | 17 | 46.155 | 41.391 | 45.728 | 40.280 |
| 36 | m | W | NA | NA | 18/09/2012 | 13 | 42.878 | 33.229 | (II) | 10.105 |
| 37 | m | W | NA | NA | 19/09/2012 | 7 | 14.122 | 9.068 | 5.464 | 3.296 |
| 52 | m | W | NA | NA | 11/09/2012 | 13 | 29.124 | 23.353 | 14.797 | 16.784 |
| 53 | m | C | 01/09/2006 | 4.76 | 18/09/2012 | 10.81 | 32.777 | 24.113 | 7.858 | 4.455 |
| 57 | m | C | 01/09/2006 | 2.52 | 11/04/2012 | 8.13 | 10.789 | 13.309 | 1.208 | 0.987 |
| 58 | m | W | NA | NA | 14/04/2012 | 5 | 0.933 | 1.725 | 0.187 | 0.000 |
